# Neoadjuvant twelve weekly paclitaxel–carboplatin with trastuzumab and pertuzumab in HER2-positive breast cancer

**DOI:** 10.1007/s10549-026-07942-4

**Published:** 2026-04-11

**Authors:** Yasmin Leshem, Inbal Golomb, Asia Zubkov, Yael Bar, Shlomit Strulov Shachar, Shir Lerner, Noa Keren-Khadmy, Amir Sonnenblick

**Affiliations:** 1 Oncology Division, Tel Aviv Sourasky Medical Center, 6 Weizmann Street, 64239 Tel Aviv, Israel; 2 Institute of Pathology, Tel Aviv Sourasky Medical Center, Tel Aviv, Israel; 3 Oncology Department, Gray Faculty of Medical & Health Sciences, Tel Aviv University, 6 Weizmann St., 6423906 Tel Aviv, Israel

**Keywords:** HER2, Breast cancer, De-escalation, Neoadjuvant therapy, Trastuzumab, Pertuzumab

## Abstract

**Purpose:**

Standard neoadjuvant therapy for early HER2-positive breast cancer consists of 18 weeks of carboplatin, docetaxel, trastuzumab, and pertuzumab. However, treatment intensity may limit feasibility in frail patients and exceed therapeutic needs in selected early-stage disease. We report here real-world clinical outcomes of patients receiving a shortened 12-week neoadjuvant regimen of weekly paclitaxel and carboplatin administered with trastuzumab and pertuzumab (12wTCHP).

**Methods:**

We conducted a retrospective analysis of patients with HER2-positive breast cancer treated with neoadjuvant 12wTCHP in a single tertiary medical center.

**Results:**

Of forty-four eligible patients receiving 12wTCHP, 41 had invasive ductal carcinoma (IDC, 93%), and 64% were ER-positive. The majority of the cohort had stage IIA (73%, median age 59 years), while the remainder had stage IIB or stage III disease and were significantly older (median age 64 and 76 years, respectively; *p* = 0.007). Grade 3–4 neutropenia (20%) and diarrhea (19%) were the most frequent toxicities. No treatment-related deaths occurred. Pathological complete response (pCR) rate was 61%: 54% in ER-positive tumors and 75% in ER-negative tumors (*p* = 0.208). After a median follow-up of 30 months, only two recurrences (5%) were observed. None of the 30 patients with stage IIA IDC had disease recurrence.

**Conclusions:**

In this retrospective cohort study, neoadjuvant 12wTCHP was well tolerated and associated with high pCR and low early recurrence rates. These findings are hypothesis-generating and support further evaluation of de-escalated 12wTCHP regimen in selected patients.

## Introduction

Over the years, the prognosis of HER2-positive breast cancer has improved dramatically with the introduction of trastuzumab and pertuzumab (HP) [1–3]. When combined with taxane and carboplatin-based chemotherapy (TCHP) as six 3-weekly cycles over 18 weeks, these agents have achieved recurrence and survival outcomes comparable to anthracycline-based regimens [4, 5], while avoiding long-term cardiotoxicity and secondary malignancies. However, even anthracycline-free protocols are associated with substantial toxicity [5], and delivering a 3-weekly regimen to elderly or frail patients is particularly challenging. Moreover, weekly taxane-based alternatives require prolonged exposure and are associated with cumulative neuropathy.

Identifying subgroups of patients who may benefit from a de-escalated treatment approach is, therefore, an area of active investigation. Among the strategies explored, a prominent example is a study by Tolaney et al., showing a very low recurrence rate in node-negative, HER2-positive breast cancers < 3 cm. This trial established a new standard of care for patients with stage I disease (< 2 cm), namely upfront surgery followed by adjuvant paclitaxel and trastuzumab (the APT regimen) [6, 7]. However, the optimal treatment approach for small T2N0 tumors, particularly those 2–3 cm in size, remains uncertain. In this setting, upfront surgery is not widely adopted due to concerns about upstaging after surgery [8], although retrospective data suggest an overall favorable prognosis [9].

In light of the excellent overall survival reported in the APT trial and the option to escalate therapy in patients who do not achieve pCR, we implemented a neoadjuvant 12-week TCHP regimen as an alternative to the standard 18-week schedule. This approach was occasionally offered to patients with small T2N0 (stage IIA) disease or those with significant comorbidities that precluded safe delivery of a 3-weekly regimen. Here we summarize our experience with 44 patients.

## Methods

### Setting and patients

This retrospective, single-center cohort study was conducted at a tertiary care hospital. We identified adult patients (> 18 years) with HER2-positive breast cancer who were prescribed neoadjuvant 12wTCHP and manually reviewed their electronic medical records. Patients were excluded if they were lost to follow-up or had received prior chemotherapy (including those who switched to 12wTCHP after starting another treatment regimen). As this is a retrospective study, no formal predefined criteria were used for treatment selection. However, in routine clinical practice, 12wTCHP was preferentially considered for patients with lower tumor burden (small T2N0 tumors) or for patients in whom the standard 3-weekly regimen was deemed less suitable due to advanced age, comorbidities, or performance status. The study was approved by the Institutional Review Board (identifier: 0215-18 TLV).

### Data review

We collected data on demographic, clinical, and pathological characteristics, Eastern Cooperative Oncology Group (ECOG) performance status along with information on toxicity, early treatment discontinuation, and dose reductions. Outcomes were last updated in December 2025.

### Treatment regimen and outcome assessment

Patients were prescribed weekly paclitaxel (80 mg/m^2^) and carboplatin (AUC 1.5 or 2) for 12 weeks, in combination with trastuzumab (8 mg/kg loading dose, followed by 6 mg/kg every 3 weeks) and pertuzumab (840 mg loading dose, followed by 420 mg every 3 weeks). After completing 12wTCHP, patients continued trastuzumab and pertuzumab until surgery. Postoperative treatment was determined by the treating physician and typically consisted of trastuzumab with or without pertuzumab in patients achieving pathological complete response (pCR), and T-DM1 in those with residual disease. Patients with estrogen receptor (ER) positive breast cancer also received adjuvant endocrine therapy.

Adverse events were graded according to the Common Terminology Criteria for Adverse Events (CTCAE) version 5. pCR was defined as no evidence of invasive disease at surgery (ypT0/TisN0). For patients with bilateral breast cancer, pCR was assigned based on the HER2-positive lesion. Recurrence-free survival (RFS) was defined as the interval from 12wTCHP initiation to the first local or distant recurrence, or to the date of last documented follow-up or death.

### Calculation of relative dose intensity (RDI)

Relative dose intensity (RDI) was calculated to reflect key modifications in chemotherapy delivery, including dose reductions, missed doses, and dose delays [10, 11]. RDI was defined as the delivered dose intensity (DDI) divided by standard dose intensity (SDI). DDI was calculated as the total delivered dose (mg/m^2^) divided by the actual treatment duration (days), with imputation for missed cycles based on the planned cycle length. SDI was calculated as the planned total dose (mg/m^2^) divided by the planned total duration (days). For example, a patient scheduled for 12 weekly doses of paclitaxel 80 mg/m^2^ (960 mg/m^2^ over 84 days), who received two full doses, followed by a 3-day delay and two reduced doses at 64 mg/m^2^ before discontinuation, had a DDI of (2 × 80 + 2 × 64) mg/m^2^ ÷ (31 days actual treatment + 56 days imputed for missed cycles). The SDI was 960 mg/m^2^ ÷ 84 days, yielding an RDI of 31%. For RDI calculation, the standard dose for carboplatin was AUC 2 per cycle.

### Statistical analysis

All statistical analyses were performed using IBM SPSS Statistics for Windows, version 25.0, or GraphPad Prism version 8.0. Descriptive statistics included medians and interquartile ranges (IQR) for continuous variables, and frequencies for categorical variables. Group differences were assessed using the Kruskal–Wallis test for continuous variables and the chi-square or two-sided Fisher’s exact test, as appropriate, for categorical variables. 95% confidence intervals (CI) for pCR proportions were calculated using the Wilson/Brown method. Recurrence-free survival was estimated using Kaplan–Meier curves.

## Results

### Patients’ characteristics

Between February 2018 and March 2025, 54 patients initiated neoadjuvant 12wTCHP, of whom 44 were eligible for analysis. Of the 10 excluded patients, five had received prior chemotherapy, three were lost to follow-up, and two were switched from a three-weekly regimen to a weekly regimen due to toxicity. Baseline characteristics are summarized in Table 1. Most patients had good performance status, with 31 (82%) having an ECOG score of 0. The median age was 65 years (IQR 52–75), and 11 patients (26%) were premenopausal. Invasive ductal carcinoma (IDC) was the predominant histology, documented in 41 patients (93%), while the remaining three (7%) had invasive lobular carcinoma (ILC). ER positivity was documented in 28 patients (63%). The median Ki-67 index was 33% (IQR 20–60). Overall, 32 patients (73%) had stage IIA (T2N0) disease, of whom 24 had tumors measuring 2–3 cm (55% of the cohort). Six patients (14%) had stage IIB disease, and the remainder had stage III disease. Patients with stage IIB and III were older (median 64 and 76 years vs. 59 years, *p* = 0.007) and had a lower rate of ECOG 0 (40% and 67% vs. 93%, *p* = 0.013), suggesting that in higher disease stages, 12wTCHP was preferentially given to older or frailer individuals. Of the 44 patients, 12 (27%) were older than 75 years. The median tumor size across the cohort was 2.6 cm (IQR 2.3–3.5) and did not vary significantly by stage.

**Table 1:** Characteristics of patients and relative dose intensity according to disease stage

	Stage IIA n = 32	Stage IIB n = 6	Stage III n = 6	p	All n = 44
Age, median (IQR)	59 (50–69)	64 (50–78)	76 (61–82)	**.007**	65 (52–75)
Premenopausal, n (%)	10 (32)	0 (0)	1 (17)	.309	11 (26)
ECOG 0, n (%)	25 (93)	2 (40)	4 (67)	**.013**	31 (82)
IDC, n (%)	30 (94)	5 (83)	6 (100)	.630	41 (93)
ER-pos, n (%)	21 (66)	4 (67)	3 (50)	.878	28 (64)
KI67, median (IQR)	33 (20–60)	30 (23–50)	40 (20–65)	.862	33 (20–60)
Tumor size in cm, median (IQR)	2.6 (2.4–3.5)	2.6 (1.5–4)	3 (2–3.6)	.758	2.6 (2.3–3.5)

Bold values indicate statistically significant results

*ECOG* Eastern Cooperative Oncology Group performance status; *IDC* invasive ductal carcinoma; *ER* estrogen receptor

### Relative dose intensity

A full paclitaxel dose (80 mg/m^2^) was prescribed in 40 patients (91%), while four started at a reduced dose. For carboplatin, 27 patients (62%) began at AUC 2 and 17 patients (39%) at AUC 1.5. Starting doses of paclitaxel and carboplatin did not significantly differ by disease stage (Table 2). The median RDI for the entire cohort was 68% (IQR 57–78). Only 10 patients (23%) achieved an RDI above 80%, whereas 24 patients (55%) received less than 70%. Patients with stage IIB and III had lower RDI compared with stage IIA (median 63% and 41% vs. 72%, respectively; *p* = 0.028). When analyzed by agent, paclitaxel RDI was significantly lower in patients with stage IIB and III compared with stage IIA (61% and 55% vs. 74%; *p* = 0.027). Carboplatin RDI was also numerically lower in stage IIB and III (65% and 20% vs. 67%; *p* = 0.062), although this difference did not reach statistical significance. These findings suggest reduced treatment tolerance in patients with more advanced stage disease, likely reflecting their older age and greater frailty.

**Table 2:** First dose and relative dose intensity (RDI) according to disease stages

	Stage IIA n = 32	Stage IIB n = 6	Stage III n = 6	p	All n = 44
Carboplatin first dose AUC 2, n (%)	19 (60)	4 (67)	4 (67)	1.00	27 (61)
Paclitaxel first dose 80 mg/m^2^, n (%)	30 (94)	5 (83)	5 (83)	.297	40 (91)
Chemotherapy RDI, median (IQR)	72 (63–80)	63 (29–72)	41 (19–62)	**.028**	68 (57–78)
Carboplatin RDI, median (IQR)	67 (53–75)	65 (18–74)	20 (16–60)	.062	65 (50–74)
Paclitaxel RDI, median (IQR)	74 (65–91)	61 (45–73)	55 (23–26)	**.027**	72 (60–88)

Bold values indicate statistically significant results

### Toxicity

As shown in Table 3, the most frequent grade 3–4 toxicities were neutropenia (20%) and diarrhea (19%). Diarrhea was the documented cause for carboplatin dose reduction in 13 patients who did not reach an RDI ≥ 80% (30% of the cohort). Grade 1–2 peripheral neuropathy was documented in 20 patients (46%), while grade 3–4 neuropathy was reported in four patients only (9%). Neuropathy was the documented cause for paclitaxel dose reduction in 14 patients who did not reach an RDI ≥ 80% (32% of the cohort). Two patients experienced febrile neutropenia (5%), and one patient (2%) had a grade 1 drop in left ventricular ejection fraction (LVEF). Seven patients (16%) required hospitalization during the treatment, including three (7%) due to grade 3 diarrhea and two (5%) due to neutropenic fever. No treatment-related deaths occurred.

**Table 3:** Treatment toxicity

	Grade 1–2 n (%)	Grade 3–4 n (%)
Anemia, n (%)	36 (90)	2 (5)
Thrombocytopenia, n (%)	11 (28)	2 (5)
Neutropenia, n (%)	15 (38)	8 (20)
Neutropenic fever, n (%)	**–**	2 (5)
Elevated liver enzymes, n (%)	19 (46)	2 (5)
Diarrhea, n (%)	25 (60)	8 (19)
Nausea, n (%)	20 (46)	0 (0)
Peripheral neuropathy, n (%)	20 (46)	4 (9)
Asthenia, n (%)	25 (66)	3 (8)
Drop in LVEF, n (%)	1 (2)	0 (0)

*LVEF* left ventricular ejection fraction

### Outcomes

**Fig. 1:**
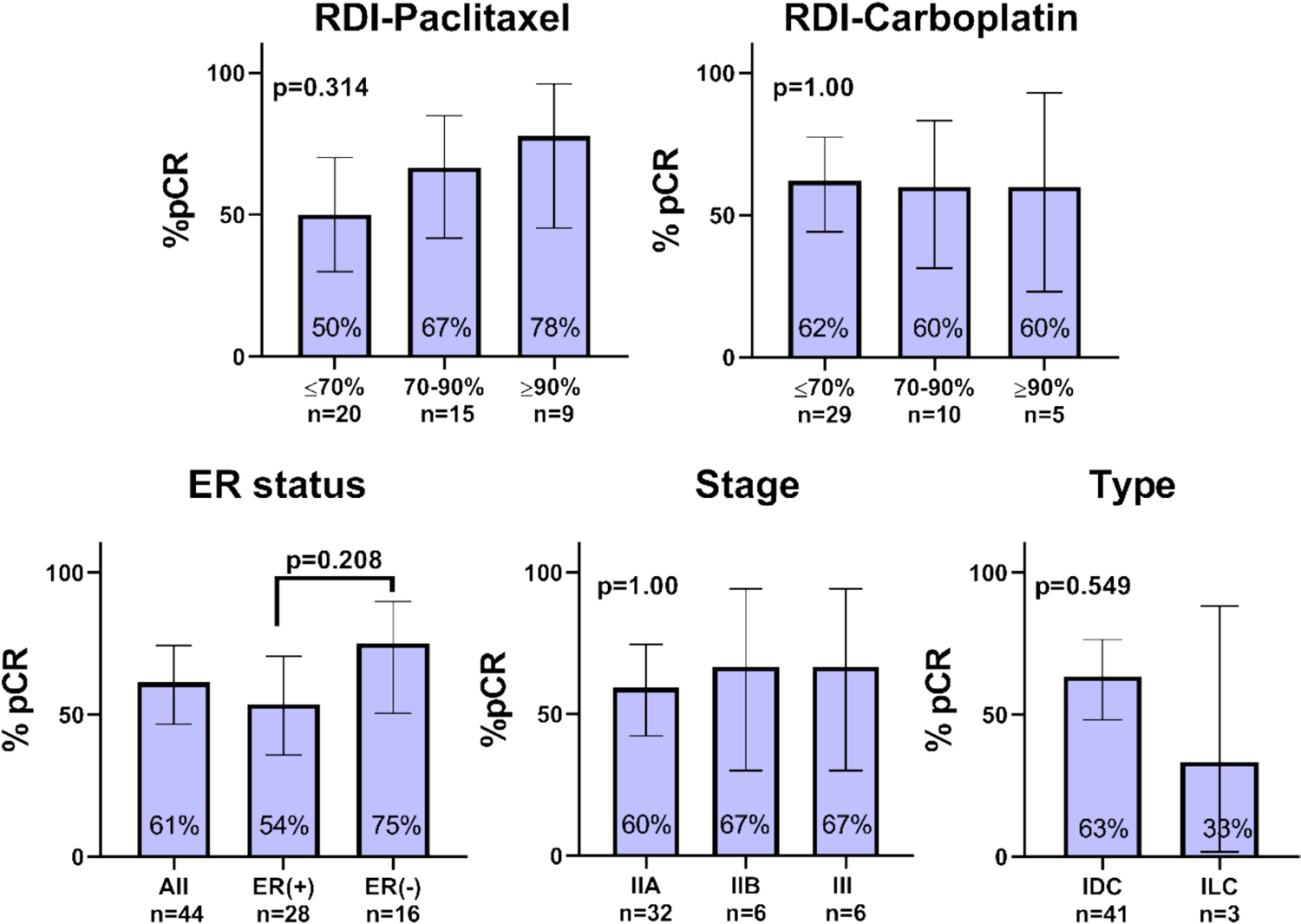
pCR rates across patient subgroups. *pCR* pathological complete response, *n* number of patients, *ER* estrogen receptor, *IDC* invasive ductal carcinoma, *ILC* invasive lobular carcinoma, *RDI* Relative dose intensity, Error bars represent 95% CI. Two-sided Fisher’s exact test was used for comparison across categories

**Fig. 2:**
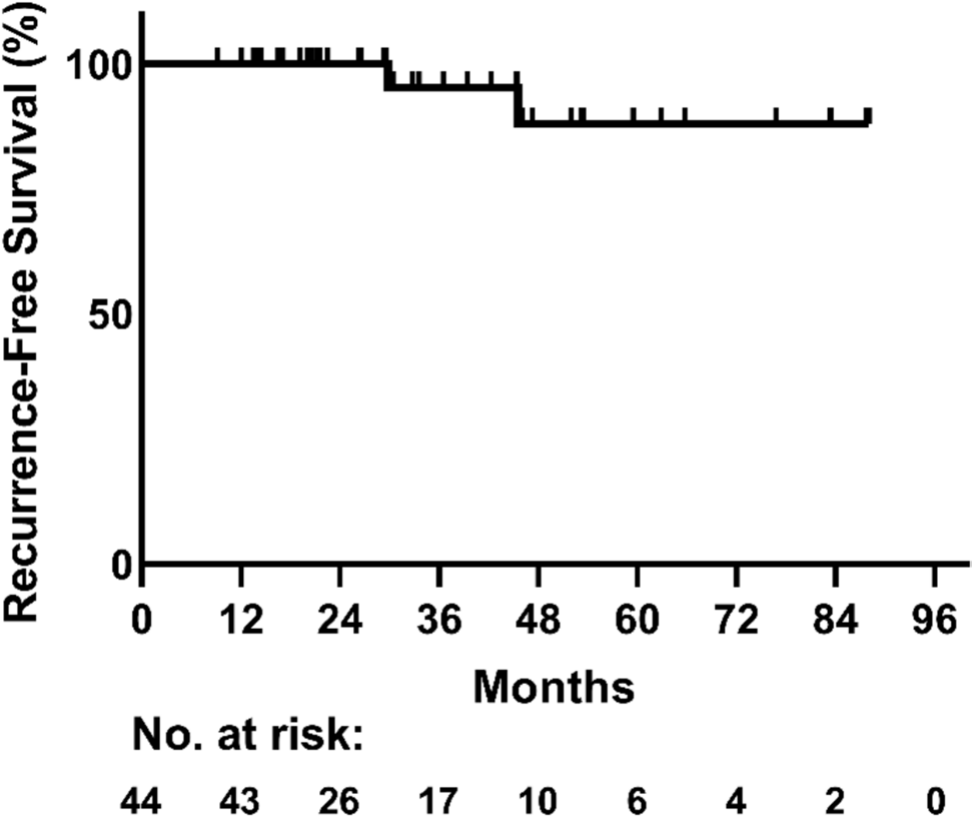
Kaplan–Meier curve for recurrence-free survival

The median follow-up was 30 months. The overall pCR rate was 61% (95% CI: 47–74). As shown in Fig. 1, 54% of patients with ER-positive tumors and 75% of those with ER-negative tumors achieved pCR (*p* = 0.208). The pCR rate was 60% in patients with stage IIA, 67% in stage IIB and 67% in stage III (*p* = 1.00). Of the three patients with ILC, one achieved pCR (33%). There was a non-significant trend toward a higher pCR rate in patients who maintained a higher paclitaxel RDI: 50% for RDI < 70%, 67% for RDI 70–90% and 78% for RDI > 90% (*p* = 0.314). No correlation was observed between carboplatin RDI and pCR. Two patients (5%) experienced a recurrence: one with stage IIA ILC, and the other with stage III IDC. Recurrence-free survival is shown in Fig. 2. Notably, none of the 30 patients with stage IIA IDC experienced a recurrence.

## Discussion

To our knowledge, this is the first study to report outcomes in patients with early-stage HER2-positive breast cancer treated with a de-escalated neoadjuvant 12wTCHP regimen. This approach was associated with a high pathological complete response (pCR) rate of 61%, a low early recurrence rate of 5%, and a favorable toxicity profile.

Treatment de-escalation for patients with early HER2-positive breast cancer has gained attention in recent years, and several strategies are currently being explored. These include shorter neoadjuvant regimens for selected patient subgroups [12] and image-guided de-escalation based on early treatment response [13, 14]. However, aside from the APT regimen, which has been widely adopted in T1N0 disease, no de-escalation strategy has gained broad consensus. The inclusion of small T2 tumors (< 3 cm) in the APT trial, along with encouraging outcomes from other studies evaluating weekly neoadjuvant regimens delivered over 12 weeks in broader patient populations [12, 15, 16] supports the rationale for adopting a de-escalated 12-week TCHP (12wTCHP) regimen in our practice. In contrast to other regimens that utilized 12 weekly cycles of neoadjuvant paclitaxel, our protocol included carboplatin (AUC 1.5–2) to better approximate the standard TCHP backbone while maintaining the shorter duration. In this retrospective cohort, treatment selection was based on physician discretion rather than predefined criteria. The cohort comprised two groups: patients with stage IIA disease (mostly tumors 2–3 cm) and older or comorbid patients with more advanced disease, in whom a de-escalated approach was considered appropriate. One major advantage of the 12wTCHP is that the weekly administration allows for close monitoring and facilitates timely modifications in response to toxicity.

The 61% pCR rate in our cohort is comparable to those reported with 18 weeks of TCHP in the TRYPHAENA (66%) [17] and TRAIN2 (67%) [18] trials, as well as in the control arms of the KRISTINE (56%) [19] and PHERGain (58%) [13] trials. Notably, our pCR rate is slightly higher than that observed in the control arm of the PREDIX (46%) [20], and PEONY (39%) [21] trials, where TCHP was administered every three weeks for 12 weeks.

Although the toxicity profile of the 12wTCHP regimen was generally favorable, grade 3–4 diarrhea occurred in 19% of patients and was the most burdensome adverse event. Despite the manageable toxicity, only 23% of patients received more than 80% of the RDI. Reduced median RDI was more prominent in carboplatin than in paclitaxel (65% vs. 72%), particularly among patients with stage III (20% vs. 55%), in whom 12wTCHP was chosen due to concerns about tolerability. These findings suggest that starting carboplatin at AUC 1.5, rather than AUC 2 may be more appropriate in frail patients.

The addition of carboplatin to paclitaxel has not demonstrated an overall survival benefit in metastatic HER2-overexpressing breast cancer [22, 23], and emerging data also question its utility in the neoadjuvant setting [24]. In our cohort, higher carboplatin RDI did not translate into improved pCR, whereas higher paclitaxel RDI was associated with a non-significant trend toward a better pCR rate. Thus, the inclusion of carboplatin in the 12wTCHP regimen may not be essential, particularly for patients with the intermediate-risk stage II disease.

Notably, the CompassHER2 trial reported a higher pCR rate with weekly paclitaxel compared to every three weeks docetaxel, raising the possibility that the favorable outcomes observed in our study are driven primarily by the weekly paclitaxel backbone [16]. However, while 12 weeks of taxane with HP (no carboplatin) has shown a remarkable pCR rate in ER-negative patients including in the ADAPT-HR-/HER2 + trial (91%) [12] and in the CompassHER2 trial (64% [16]), the pCR rate in ER-positive subpopulation was reported to be lower (32% [16]). These findings suggest that carboplatin may be dispensable in ER-negative disease, while additional data are needed to define its role in ER-positive tumors.

Our cohort included three patients with ILC, of whom only one achieved a complete response. Notably, the only recurrence within stage IIA subgroup occurred in a patient with ILC. However, given the very small number of ILC cases, these findings are hypothesis-generating, should be interpreted with caution, and no firm conclusions can be drawn. None of the 30 patients with stage IIA IDC experienced recurrence, supporting further investigation of the 12w TCHP regimen for T2N0 HER2-positive IDC.

Our study has several inherent limitations. First, its retrospective design and single-center nature may limit the generalizability of the findings. Second, patients were selected for 12wTCHP based on physician discretion, which introduces selection bias. Importantly, the small sample size (n = 44) substantially limits statistical power, particularly for subgroup analyses by ER status, stage, and histology and therefore, these analyses should be considered exploratory and interpreted with caution. Additionally, the follow-up period may be insufficient to capture the true recurrence rate, particularly in ER-positive disease.

Altogether, this retrospective cohort suggests favorable efficacy and tolerability of neoadjuvant 12wTCHP. These hypothesis-generating findings warrant prospective validation and further study to define its role in selected patients, including those with stage IIA disease or vulnerable populations.

## Data Availability

The data underlying this article will be shared on reasonable request to the corresponding author.
